# Introducing Discrete Frequency Infrared Technology for High-Throughput Biofluid Screening

**DOI:** 10.1038/srep20173

**Published:** 2016-02-04

**Authors:** Caryn Hughes, Graeme Clemens, Benjamin Bird, Timothy Dawson, Katherine M. Ashton, Michael D. Jenkinson, Andrew Brodbelt, Miles Weida, Edeline Fotheringham, Matthew Barre, Jeremy Rowlette, Matthew J. Baker

**Affiliations:** 1 University of Manchester, School of Chemical Engineering and Analytical Science, Manchester, M13 9PL, United Kingdom; 2 WestCHEM, Technology and Innovation Centre, 99 George St, Department of Pure and Applied Chemistry, University of Strathclyde, Glasgow, G1 1RD, UK; 3 Daylight Solutions Inc., San Diego, CA 92128, USA; 4 Lancashire Teaching Hospitals NHS Trust, Royal Preston Hospital, Department of Pathology, Preston, PR2 9HT, UK; 5 The Walton Centre NHS Foundation Trust, The Walton Centre for Neurology and Neurosurgery, Liverpool, L9 7LJ, UK

## Abstract

Accurate early diagnosis is critical to patient survival, management and quality of life. Biofluids are key to early diagnosis due to their ease of collection and intimate involvement in human function. Large-scale mid-IR imaging of dried fluid deposits offers a high-throughput molecular analysis paradigm for the biomedical laboratory. The exciting advent of tuneable quantum cascade lasers allows for the collection of discrete frequency infrared data enabling clinically relevant timescales. By scanning targeted frequencies spectral quality, reproducibility and diagnostic potential can be maintained while significantly reducing acquisition time and processing requirements, sampling 16 serum spots with 0.6, 5.1 and 15% relative standard deviation (RSD) for 199, 14 and 9 discrete frequencies respectively. We use this reproducible methodology to show proof of concept rapid diagnostics; 40 unique dried liquid biopsies from brain, breast, lung and skin cancer patients were classified in 2.4 cumulative seconds against 10 non-cancer controls with accuracies of up to 90%.

Key objectives for new diagnostic systems for diseases such as cancer include improving patient outcome through the identification of earlier stages, monitoring drug resistance and identifying high-risk populations for tumour progression. New diagnostic technological advances are not only required to be highly reliable but must also conform to cost-effective and high-throughput standards due to the drivers of ever-increasing workloads and rising costs in the medical profession.

Mid-infrared spectroscopy harnesses infrared radiation in the ν 25–120 THz frequency range (λ 2.5–12 μm, 1/λ 4000–830 cm^−1^) of the electromagnetic spectrum. The technique enables global bio-molecular fingerprints of complex organic materials to be recorded in a non-destructive and stain-free process^1^.

Attenuated total reflection (ATR-FTIR) spectroscopy is highlighted as an appropriate approach for point-of-care applications due to the minimal sample preparation and ability to rapidly acquire a single sample spectrum (in the order of seconds). While the method has been proven to provide cancer discrimination using blood serum^2^, it does not offer high-throughput utility as it requires a liquid biopsy to be spotted and dried on the ATR crystal surface before the spectrum is recorded. The drying process can take several minutes and the crystal must be subsequently cleaned before a new sample is presented; as a consequence, storage of samples for repeat measurements is also not an option^3^.

Mid-infrared Fourier transform micro-spectroscopic (FTIRM) imaging has been suitably applied to compliment histological, cytological and pathological studies to detect subtle changes in the proteome and metabolome of diseased cells and tissue. FTIRM is fast proving to be a reliable research tool with clinical utility for disease-state diagnostics^4^,^5^,^6^,^7^,^8^. The application of IR imaging for dried liquid blood biopsy serum samples, however, is a relatively new application^3^,^9^. By using an infrared imaging approach, a high-throughput screening application can be realised for the biomedical laboratory; liquid biopsies that are dispensed and dried to create multi-patient micro-arrays to deliver rapid data collection, quality control and classification processes^3^.

Biomedical applications of FTIRM have been proved in research laboratories but despite a surge in technological advance and theoretical understanding^10^, FTIRM has yet to be fully integrated into large-scale clinical application. One of the technological caveats of the imaging technique is the requirement to scan the entire IR spectrum; a redundant approach as clinically relevant diagnostic markers are found at very specific frequencies. The infrared source on a benchtop FTIRM system is also low-throughput and can require longer scanning times to improve signal to noise. As a consequence, FTIRM in its current state of development is not considered to be an efficiently rapid and high-throughput technique per se.

The recent integration of high-brightness, broadly tuneable mid-infrared quantum cascade lasers (QCLs) into a microscope system may address these concerns. The combination of the QCL source, refractive-based high numerical aperture objectives and a large format detector system has enabled the option of high-definition diffraction-limited resolution, without a trade-off in signal to noise and field of view when compared to FTIR-based microscope systems. The technique is called discrete frequency infrared (DFIR) imaging as the laser is tuneable to a custom range, providing a substantial reduction in data acquisition time, creating potential for efficient, real-time data collection for disease-specific diagnostics on a clinically appropriate time scale^11^,^12^.

Results presented in this communication illustrate the developmental application and validation of DFIR with a focus on cancer detection using human blood serum. Initially, the use of an automated piezo-jetted serum dispenser and subsequent DFIR imaging of dried serum spots from human pooled serum are assessed to explore sample uniformity. The samples were imaged using different discrete frequency ranges and subsequently assessed after implementing data processing and quality control mechanisms. The advantage of rapid infrared imaging as opposed to point spectra is the ability to evaluate and monitor sample quality control requirements. Secondly, it was necessary to investigate whether the diagnostic capability of cancer detection was preserved when moving from full frequency (as would be collected using an FTIR system) to discrete frequency mode (resulting in a reduction in spectral information). Finally, the high-throughput diagnostic capability of DFIR technology is presented by a proof of concept example involving a series of samples from multiple patients presenting with different cancers.

## Results

### Investigating collection parameters and sample reproducibility

Several studies have proven manual spotting of serum samples to be an adequate method of preparation for disease-state IR diagnostics^2^,^13^,^14^. In a clinical setting, however, an automated dispenser would need to be utilised to increase consistency in sample placement, volume accuracy and high-throughput sample processing as recent investigations have shown^15^. A total of 40 technical replicate serum spots from human pooled serum were used to investigate the feasibility of automated sample preparation and also to determine the consequence of DFIR parameter selection on spectral reproducibility.

**Figure 1:**
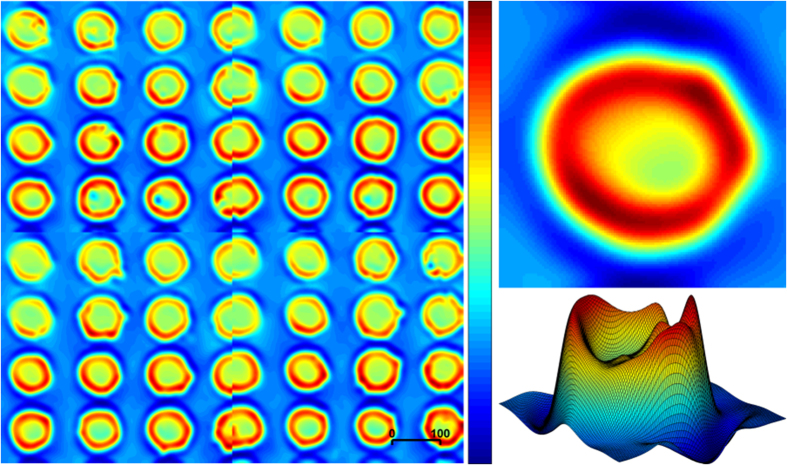
Technical replicability of automated liquid biopsy dispensing. Automated sample preparation of 56 dried biopsies from pooled human blood serum; A 2 × 2 mosaic DFIR image displaying total intensity of infrared absorbance which indicates sample thickness (with increasing values from blue to red as shown in the colour bar). The automated dispenser allowed for the creation of smaller deposits relative to hand-dispensed samples; the scale bar displays 100 pixels (equivalent to a size of 130 μm). An individual sample is also displayed in a 2D and topographical mesh diagram showing uneven sample thickness, confirming the ‘coffee-ring’ drying phenomenon. The sample was imaged three times using 199, 14 and 9 discrete frequencies.

The serum spots displayed fairly consistent morphological drying patterns, as illustrated by their 3D mesh plots displaying total intensity of infrared absorbance (Fig. 1). The samples were chemically imaged in a 2 × 2 mosaic map using three different DFIR ranges at either 199, 14 or 9 specific frequencies (Table 1). Each replicate image was subject to a process of quality control by spatially locating pixel spectra of poor quality, which resulted from issues relating to sample thickness, signal to noise and scattering. For each replicate, the remaining spectra were averaged, creating a single high-quality spectrum per sample.

**Figure 2:**
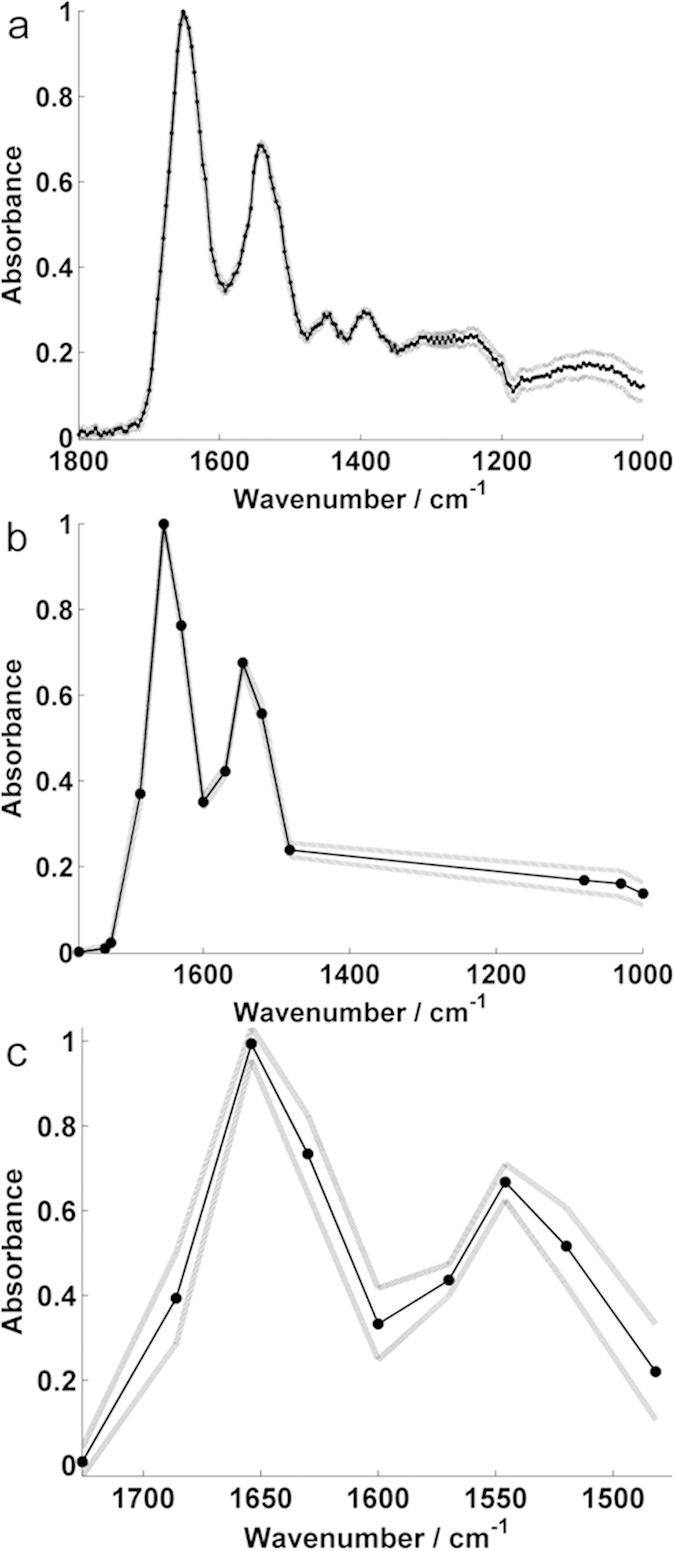
Data processing replicability for different data collection frequencies. Samples shown in Fig. 1 were imaged using different frequency ranges. A total of 40 of the technical replicate deposits were subject to individual data processing for each frequency range. Mean representative spectra from the 40 technical replicates ± standard deviation are shown for (**a**) 199 (**b**) 14 and (**c**) 9 discrete frequencies.

The mean variability for each dataset of the 40 replicate observations is shown in Table 1 and Fig. 2. The impact of the quality control regime and frequency range on spectral reproducibility indicated that the use of 14 discrete frequencies, chosen from the fingerprint range, provided the best compromise between speed of acquisition and reproducibility.

### Moving from full to sparse frequency: Diagnostic potential is maintained

Non-cancerous serum samples have previously been discriminated from patients with brain tumours of increasing severities in low-grade to high grade gliomas with the use of ATR-FTIR spectroscopy and chemometric analytical techniques; Cancerous and non-cancerous blood serum could be classified with sensitivities and specificities of 94 and 97% respectively^2^. The discriminatory spectral features between the non-cancer and cancerous samples were found at frequencies largely associated with the amide I and II bands which correspond to C=O stretching, C-N stretching and N-H bending vibrational modes^9^.

**Figure 3:**
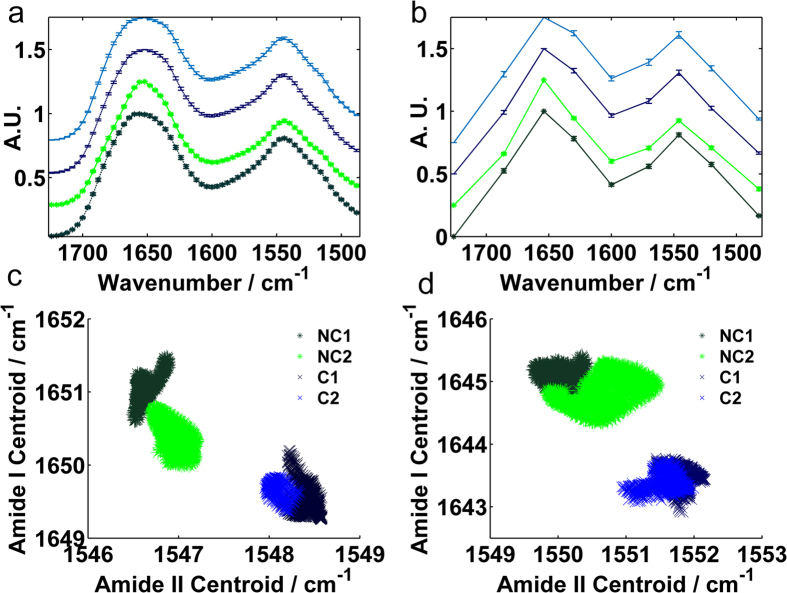
Comparing diagnostic potential from full range to sparse frequency data. (**a**,**b**) mean representative spectra for non-cancer (NC, green, *) and brain cancer (C, blue) patient samples. Error bars denote standard deviation (mean values of standard deviation for NC1, NC2, C1, C2 were 0.01, 0.009, 0.009, 0.008 for full and 0.011, 0.010, 0.012, 0.016 for sparse range respectively. (**c**,**d**) Corresponding Amide I and II peak centroid correlation plots for full and sparse frequency data collection modes respectively.

In order to investigate whether the same discriminatory features could be found in the serum using rapid DFIR technology, serum samples from brain were analysed by using a quantum cascade laser (QCL) IR system for both full-frequency and discrete-frequency (DFIR) modes (specifically 14 frequencies in this case). Figure 3a,b display the mean representative spectrum recorded from serum samples of non-cancerous (green) and cancerous (blue) patients using full and sparse frequency data collection modes respectively. Despite the substantial reduction in data, the discrete frequency spectra exhibited recognisable bandshapes of their equivalent full frequency spectra.

Centroid position values were calculated for the amide I and II peaks and subsequently displayed by the ratio in the correlation plots in Fig. 3c,d for full and sparse frequency modes respectively, where differences were revealed; For the discrete frequency data, the average peak centroid values had shifted relative to the full frequency positions (1651 to 1645 cm^−1^ and 1548 to 1552 cm^−1^ for the amide I and II peaks respectively). Inevitably, there was greater sensitivity in the intra-class distinction using the full data range mode, as data points belonging to individual patient sample spectra can be more clearly resolved. This is due to the higher accuracy in peak centroid calculation as a result of the additional data points contributing to peak band shape.

It was noted, however, that both full and discrete frequency modes resulted in incontestable discrimination of cancer versus non-cancer data overall, demonstrating that streamlined data acquisition via discrete frequency mode has an equally acceptable diagnostic power in the goal of rapid cancer detection. There are currently no techniques in the clinic for the discrimination of cancer vs non-cancer for multiple disorders. To compare and benchmark our results a study on breast mammography using 2 radiologists (240 women with breast cancer and 240 women with normal breast) reported sensitivities and specificities of 75.8 and 85.4% for radiologist 1 and 75.4 and 89.2% for radiologist 2 respectively^16^. This demonstrates that our results are of clinical acceptance, can be provided before and help direct an invasive procedure, but are also a direct physical measurement of the patient blood biochemistry. Conventional methods are reliant upon human interpretation of tissue biopsy morphology and thus often provide more sporadic levels of inter-observer accuracy within the clinic and can be inadequate to flag the early signs of brain disease. It is quite often the case that by the time symptomatic changes are addressed via imaging and biopsy, the disease is at an advanced stage and prognosis is poor. Thus, a serum based screening via infrared methods may in the future help elucidate earlier forms of disease and allow more successful intervention and treatment.

### Demonstrating proof of concept for DFIR rapid cancer detection

Using the results of the preliminary investigation and knowledge from previous studies, an unprecedented proof of concept experiment was conducted to test the premise that DFIR imaging technology could rapidly and consistently lead to the correct classification of dried fluid-biopsies from multiple individuals that presented with different cancers.

A sample matrix of 50 human blood serum liquid biopsies were prepared by spotting onto a single slide and subsequently air-dried. The sample matrix was comprised of 10 individual observations from non-cancer controls, and brain, breast, lung and skin cancer. The sample was imaged as one DFIR image mosaic. For each patient, a single representative spectrum comprising of just 14 discrete frequencies was obtained.

The spectra were then tested for cancer detection using a radial-basis-function support vector machine (SVM) model^3^,^14^. Each cancer spectrum in its class was tested against a model built on a population of remaining cancer patient spectra and all control spectra. Each of the 40 cancer patient spectra were tested in triplicate, with slight variations in model parameters in the different iterations (SI 1). Each time the SVM model was trained, a single patient spectrum took 0.02 seconds to test. In almost all cases, the classification model was robust; over the 3 replicate testing runs, a unanimous decision was reached 95% of the time. For the two instances which resulted in different decision outcomes when tested in the triplicate, the modal outcome was taken (SI 1).

**Figure 4:**
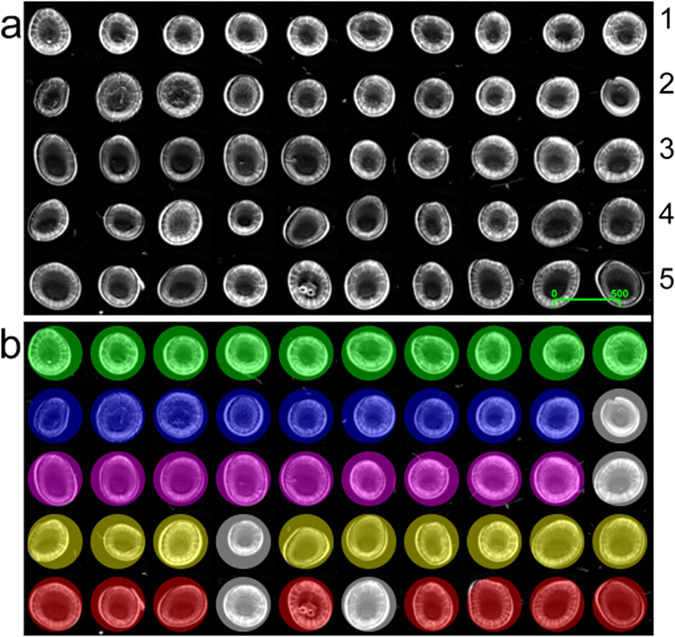
Rapid classification of multiple cancers using dried human serum liquid biopsies. (**a**) Grayscale image mosaic of 50 unique serum biopsies (row *1* non-cancer controls, *2* brain *3* breast, *4* lung and *5* skin cancer patient samples) the scale bar displays 500 pixels (equivalent to a size of 2.1 mm). (**b**) Classification outcomes for correct cancer diagnosis were accurate to 90%, 90%, 90% and 80%, highlighted by colour for brain (blue), breast (pink), lung (yellow) and skin (red) cancer samples respectively.

The concluding ‘cancer versus non cancer’ classification test had a 9/10 success rate for brain, breast and lung patients and an 8/10 rate for skin cancer patients (Fig. 4). It took a cumulative time of 2.4 seconds to test (in triplicate) the 40 cancer-patient samples.

## Discussion

As with any new approach, it is important to consider parameter selection in order to achieve quality data, collected with maximum efficiency. One can see from Fig. 1 that even serum droplets created with an automated dispenser can exhibit the so-called ‘coffee-ring effect’^3^. The drying pattern of the serum can cause variation in sub-regional sample thickness and molecular composition, as well as causing physical light scattering perturbations that can contaminate the spectral signature of the sample^3^. Any difference in molecular composition across the sample can be managed by averaging the spectral data collected in each pixel associated with an individual serum spot. The spectral perturbations caused by influence of light scattering from a bio-fluid sample are far from acute, in comparison to biological cells for instance. Nevertheless, it is important to remove such affected spectra and spectra dominated by saturation due to non-linear absorption from the overall sample measurement.

The initial experiment compared the findings taken with both discrete frequency and full frequency (‘FTIR-like’) approaches from a small sample batch consisting of hundreds of pixel spectra. Despite the fact that a quality control test was not adopted at this stage (spectra were randomly selected from the centre of the serum spot images), both spectral datasets were in agreement; the scatter pattern of amide I/II centroid ratios convincingly distinguished cancer from non-cancer control data points. The pattern indicated that subtle changes in the proteome of the complex serum bulk (on a macroscopic scale) would be detected whether full or discrete frequency data collection mode was chosen.

A proof of concept study was performed to illustrate DFIR-led rapid diagnosis of individual fluid biopsies in multiple cancers. A mosaic DFIR hyperspectral image was constructed with 50 unique serum spot samples. The spectral outputs for the mosaic were systematically assessed under a quality control strategy before the representative spectrum of the individual serum spot was produced. Each cancer patient sample was blind-tested against a model built from respective cancer patient class data alongside control samples.

The testing process for a patient spectrum was performed with almost instantaneous speed at 0.02 seconds. It is true that the 93% reduction of number of required data points is certainly a factor in the classification speed (i.e. 14 data points compared to a full frequency spectrum equivalent (of the fingerprint range) with 199 data points). This is not, however, a result to champion in isolation given that a full frequency single spectrum would still be arguably very fast to classify.

One major advantage of acquiring DFIR data lies in the lighter computational requirements for pre-processing prior to the creation of single spectra. A simple method to improve big data management is to reduce the initial data set size. The mosaic image shown in Fig. 4 with 50 spots has dimensions of 2400 × 4800 pixels; the unprocessed hyperspectral image will contain 1.344 × 10^7^ spectra. With only 14 discrete frequencies in DFIR mode, the total number of data points sampled would equate to 1.882 × 10^8^ (as a point of measure, the 14-DFIR mosaic image loaded into Matlab with 1.882 × 10^8^ data points required 1.83 GB of memory). If the image was created using a wider frequency range, such as 199 frequencies, this would increase to 2.675 × 10^9^ data points. Increasing the number of frequencies sampled therefore has a considerable effect on computational power and inevitable implications on speed during the spectral pre-processing and quality control steps.

The second advantage is the actual time taken to acquire the data. Using the collection parameters as described in the methods section, a single image of a single serum spot would take 11.3 minutes for 1000–1800 cm^−1^ (199 data points), 1.7 minutes for 14 data points and 1 minute for 9 data points. (NB: There have already been recent advances made in the speed of data collection since these experiments were conducted; the laser can now be programmed to execute a sweep of wavelengths, sending triggers to the software to acquire frames when it has reached each wavelength, which is a much more efficient method to acquire the data and has subsequently reduced the time taken for the 199 data points to 4.3 minutes, for 14 points to 56 seconds and 9 points to 36 seconds).

Upon consideration of these results, further investigation of technical parameters and optimisation in sample preparation was considered. The use of an automated piezo dispensing system allowed for more consistency and produced smaller spots with a more consistent size due to spotting accuracy and volume control. As a consequence, this would also increase the high-throughput spot capacity as a result of the smaller sample spot area.

Considerable reduction of spectral acquisition time was possible by using 14 discrete frequencies (Table 1). The spots all originated from the same patient sample. In terms of intra-sample variability, despite the 4.5% increase in relative standard deviation, the average change in the overall spectral absorbance remained consistent with the full frequency range. The largest region of spectral variance resided in the lower frequency region (towards 1000 cm^−1^). For spectral baseline purposes, it was useful to collect one or two data points at these lower frequencies. Once spectra have been scaled, however, variability can be reduced further by subsequent spectral truncation; especially relevant if the most salient features for the diagnostic test were found in the higher frequency (amide I and II) regions.

To meet high-throughput requirements of a clinical diagnostic tool, we have shown that automated sample preparation can increase technical replicate consistency and conformity, maximising the number of samples probed in the sampling area. In terms of data processing and accessibility, spectral apps are also currently in development that would allow mobile devices to access and manipulate spectra for real-time reporting^17^.

Given the full frequency studies have been thoroughly conducted, transition to DFIR is an obvious step towards building a rapid disease detection tool. By tuning the QCL to acquire data from frequencies of interest, DFIR imaging allows the opportunity to significantly increase the speed of data acquisition and processing. The indicative power of the three studies combined demonstrate the solid proof of concept that DFIR imaging is a plausible technological candidate to achieve a rapid, high-throughput tool for cancer diagnostics in the near future.

## Methods

### Ethical Approval

Human blood serum patient samples were obtained from the Walton Research Tissue Bank (WRTB) and Brain Tumour North West (BTNW) Tissue Bank with informed consent from all subjects. Joint application for clinical samples for experiments performed under guidelines and regulations from the Brain Tumour North West and the Walton Research Tissue Banks were approved by the BTNW/WRTB Committee (BTNW/WRTB 13_01, Application Number 13_01). Samples obtained represent high-grade gliomas (primary brain tumours) (WHO Grade III and IV) and skin, breast and lung tumours that have metastasised to the brain.

### Sample preparation

Pooled human serum was purchased from TCS Biosciences, UK (0.2 μm sterile filtered CS100–100). 56 technical replicate spots were dispensed using a piezo-driven auto-dispenser every 6.0 ms at the speed of 100 mm/sec and were left to dry in ambient conditions. The human serum of tissue bank origin was manually pipetted by hand in 0.5 μL droplets and left to dry in ambient conditions.

### Data collection

All spectral data was acquired using a QCL Spero™ microscope (Daylight Solutions Inc., San Diego, CA, USA). All data supporting this research are openly available from MJB. The authors direct the readers to Hughes *et al*.^18^ for a published schematic of the instrument. Data was acquired in different ranges; either at 199, 14 or 9 frequencies (specific frequencies can be found in Table 1). The specific frequencies were chosen based upon the study by Hands *et al*.^2^. All data collected during this study was recorded using what we term, “Step and Measure Mode”, which is software driven. Briefly, the laser is commanded to step to a wavelength and an intensity image is acquired. Multiple frames can be acquired to increase signal-to-noise for low light level situations. The software then commands the laser to tune to the next wavelength in an iterative process.

The piezo-jetted drops were acquired using a 2 × 2 mosaic with a high magnification objective (Fig. 1) and were ca. 100 μm in diameter. (FOV = 650 μm × 650 μm, 12.5 × magnification, NA = 0.70, pixel size = 1.36, spatial resolution = ca. 5 μm at λ 5.5 μm). The hand dropped serum spots were ca. 1000 μm in diameter acquired using single frames with a low magnification objective (FOV = 2 mm × 2 mm, 4 × magnification, numerical aperture (NA) = 0.15, pixel size = 4.25, spatial resolution = ca. 25 μm at λ 5.5 μm). The single frames created 5 × 10 mosaic matrix shown in Fig. 3.

### Data processing and analysis

For the initial experiment, serum spots were considered on an individual basis. The 56 serum spots were captured in a 2 × 2-frame mosaic IR image. A total of 40/56 spots were analysed for reproducibility, as some of the spots were not completely captured, or fell on the boundaries when the single title images were stitched together (Fig. 1). Mean spot spectra were generated after the quality control process as described for the second experiment. Reproducibility was assessed by determining both the average change in absorbance and average relative standard deviation across each frequency range for the 40 spectral observations (Table 1, Fig. 2).

In order to compare the full and discrete frequency data, spectra were sampled from the centre of two brain cancer patient and two control serum spots that were manually spotted. Data was collected with both full and discrete frequency ranges (199 or 14 data points) and was subject to the same processing treatment by min-max scaling. The two datasets were compared by determining the peak centroid spectral moment for the amide bands in each individual spectrum, where differences in band frequency position and band shape will determine the centroid value. The centroid positions of the amide I and amide II band were correlated, illustrated in Fig. 1(c,d).

In the final experiment, a multi-mosaic image was constructed that comprised of 50 manually prepared serum spots. The spots represented five different classes; non-cancer controls versus, brain, breast, lung and skin cancer; all sample spots were sourced from 50 independent patient subjects. Image segmentation was applied using *k*-means cluster analysis to identify good quality spectra from each pixel that was not representative of either background (no or low signal) or distorted spectra (saturated data due to sample thickness) (SI 2). Quality-passed spectra were averaged to create a single representative spectrum per patient sample. A radial-basis function support vector machine (RBF-SVM) Model was used to demonstrate non-cancer/cancer proof of concept classification. Classification was done on a separate basis for each cancer class versus the non-cancer control set. The model was trained with the 10 control spectrum plus 9 of the cancer class spectra on a ‘leave-one-out’ basis, i.e. to test brain cancer patient 1 data, the model was training using patients 2–10. The training included 3-fold cross-validation to determine the SVM parameters (C and γ) to gain the best training classification accuracy. The parameter values obtained for patient 1 were then fixed when repeating the training model to test patients 2–10 respectively, which resulted in either positive or negative classification. For robustness, each overall cancer model was trained in triplicate as the SVM parameters could be subject to value changes (SI 1).

## Additional Information

**How to cite this article**: Hughes, C. *et al*. Introducing Discrete Frequency Infrared Technology for High-Throughput Biofluid Screening. *Sci. Rep*. **6**, 20173; doi: 10.1038/srep20173 (2016).

## Supplementary Material

Supplementary Information

## Figures and Tables

**Table 1:** Data collection speeds and spectral reproducibility for the mosaic image (Figure 1) acquired using three different DFIR ranges.

**Number of Discrete Frequencies**	Discrete Frequencies*	Acquisition Time** **(min)**	**∆Abs**+	**RSD**++ (**%)**
199	1000–1800	45.2	0.017	0.6
14	1000, 1030, 1080, 1482, 1520, 1546, 1570, 1600, 1630, 1654, 1686, 1726, 1734, 1770	6.8	0.017	5.1
9	1482, 1520, 1546, 1570, 1600, 1630, 1654, 1686, 1726	4.0	0.071	15.0

^*^Quoted in wavenumbers (cm^−1^). The 1000–1800 cm^−1^ range included data point spacing of 4 cm^−1^.

^**^The data collection time for the (2 × 2 tile) image.

^+^The average change in absorbance value across the discrete frequencies for the spectral dataset (n = 40).

^++^The mean relative standard deviation for the spectral dataset (n = 40).
