# Supplementary material for: Introducing Discrete Frequency Infrared Technology for High-Throughput Biofluid Screening
Source: Sci Rep. 2016 Feb 4;6:20173. doi: 10.1038/srep20173 (PMC4740754; doi:10.1038/srep20173)

Introducing Discrete Frequency Infrared Technology for High-Throughput Biofluid Screening

Supplementary Information

Caryn Hughes[1, 2*], Graeme Clemens[2], Benjamin Bird[3], Timothy Dawson[4], Katherine M. Ashton[4], Michael D. Jenkinson[5], Andrew Brodbelt[5], Miles Weida[3], Edeline Fotheringham[3], Matthew Barre[3], Jeremy Rowlette[3] and Matthew J. Baker[2*]

[1] University of Manchester, School of Chemical Engineering and Analytical Science, Manchester, M13 9PL, United Kingdom

[2]University of Strathclyde, Department of Pure and Applied Chemistry, Glasgow, G1 1XL, UK

[3] Daylight Solutions Inc., San Diego, CA 92128, USA

[4] Lancashire Teaching Hospitals NHS Trust, Royal Preston Hospital, Department of Pathology, Preston, PR2 9HT, UK

[5] The Walton Centre NHS Foundation Trust, The Walton Centre for Neurology and Neurosurgery, Liverpool, L9 7LJ, UK

[*]Corresponding Authors: caryn.hughes@manchester.ac.uk @Dr_Caryn_Hughes; matthew.baker@strath.ac.uk, @ChemistryBaker

Keywords: Imaging, Discrete Frequency Infrared Spectroscopy (DFIR), Biomedical, Quantum Cascade Laser (QCL)

Contents

SI 1: Training parameters and classification outcomes 2

SI 2: Quality control regime for the generation of single biopsy spectra 6

SI 1: Training parameters and classification outcomes

(a) Image segmentation was applied using *k*-means cluster analysis to identify good quality spectra from each pixel that was not representative of either (b) background (no or low signal, class 2) or distorted spectra (saturated data due to sample thickness class 3). Quality-passed spectra were averaged to create a single representative spectrum per patient sample (class 1).

Table SI 1.1

BRAIN CANCER<br>TRAINING PATIENTS	TEST PATIENT	TRAINING RUN 1				TRAINING RUN 2				TRAINING RUN 3			
		% Training Accuracy	C	γ	Correct Classification	% Training Accuracy	C	γ	Correct Classification	% Training Accuracy	C	γ	Correct Classification
2 3 4 5 6 7 8 9 10	1	94.7	2048	0.5	✓	94.7	2048	0.088	✓	94.7	2048	0.5	✓
1 3 4 5 6 7 8 9 10	2				✓				✓				✓
1 2 4 5 6 7 8 9 10	3				✓				✓				✓
1 2 3 5 6 7 8 9 10	4				✓				✓				✓
1 2 3 4 6 7 8 9 10	5				✓				✓				✓
1 2 3 4 5 7 8 9 10	6				✓				✓				✓
1 2 3 4 5 6 8 9 10	7				✓				✓				✓
1 2 3 4 5 6 7 9 10	8				✓				×				✓
1 2 3 4 5 6 7 8 10	9				✓				✓				✓
1 2 3 4 5 6 7 8 9	10				×				×				×

Table SI 1.2

BREAST CANCER<br>TRAINING PATIENTS	TEST PATIENT	TRAINING RUN 1				TRAINING RUN 2				TRAINING RUN 3			
		% Training Accuracy	C	γ	Correct Classification	% Training Accuracy	C	γ	Correct Classification	% Training Accuracy	C	γ	Correct Classification
2 3 4 5 6 7 8 9 10	1	94.7	64	2.83	✓	94.7	64	1.19	✓	94.7	64	2.83	✓
1 3 4 5 6 7 8 9 10	2				✓				✓				✓
1 2 4 5 6 7 8 9 10	3				✓				✓				✓
1 2 3 5 6 7 8 9 10	4				✓				✓				✓
1 2 3 4 6 7 8 9 10	5				✓				✓				✓
1 2 3 4 5 7 8 9 10	6				✓				✓				✓
1 2 3 4 5 6 8 9 10	7				✓				✓				✓
1 2 3 4 5 6 7 9 10	8				✓				✓				✓
1 2 3 4 5 6 7 8 10	9				✓				✓				✓
1 2 3 4 5 6 7 8 9	10				×				×				×

Table SI 1.3

LUNG CANCER TRAINING PATIENTS	TEST PATIENT	TRAINING RUN 1				TRAINING RUN 2				TRAINING RUN 3			
		% Training Accuracy	C	γ	Correct Classification	% Training Accuracy	C	γ	Correct Classification	% Training Accuracy	C	γ	Correct Classification
2 3 4 5 6 7 8 9 10	1	94.7	362.04	16	✓	100	362.04	2.82	✓	94.7	64	16	✓
1 3 4 5 6 7 8 9 10	2				✓				✓				✓
1 2 4 5 6 7 8 9 10	3				✓				✓				✓
1 2 3 5 6 7 8 9 10	4				×				✓				×
1 2 3 4 6 7 8 9 10	5				✓				✓				✓
1 2 3 4 5 7 8 9 10	6				✓				✓				✓
1 2 3 4 5 6 8 9 10	7				✓				✓				✓
1 2 3 4 5 6 7 9 10	8				✓				✓				✓
1 2 3 4 5 6 7 8 10	9				✓				✓				✓
1 2 3 4 5 6 7 8 9	10				✓				✓				✓

Table SI 1.4

SKIN CANCER TRAINING PATIENTS	TEST PATIENT	TRAINING RUN 1				TRAINING RUN 2				TRAINING RUN 3			
		% Training Accuracy	C	γ	Correct Classification	% Training Accuracy	C	γ	Correct Classification	% Training Accuracy	C	γ	Correct Classification
2 3 4 5 6 7 8 9 10	1	89.5	26.91	0.21	✓	89.5	64	0.21	✓	89.5	26.91	0.5	✓
1 3 4 5 6 7 8 9 10	2				✓				✓				✓
1 2 4 5 6 7 8 9 10	3				✓				✓				✓
1 2 3 5 6 7 8 9 10	4				×				×				×
1 2 3 4 6 7 8 9 10	5				✓				✓				✓
1 2 3 4 5 7 8 9 10	6				×				×				×
1 2 3 4 5 6 8 9 10	7				✓				✓				✓
1 2 3 4 5 6 7 9 10	8				✓				✓				✓
1 2 3 4 5 6 7 8 10	9				✓				✓				✓
1 2 3 4 5 6 7 8 9	10				✓				✓				✓

SI 2: Quality control regime for the generation of single biopsy spectra

(a) Image segmentation was applied using *k*-means cluster analysis to identify good quality spectra from each pixel that was not representative of either (b) background (no or low signal, class 2) or distorted spectra (saturated data due to sample thickness class 3). Quality-passed spectra were averaged to create a single representative spectrum per patient sample (class 1).

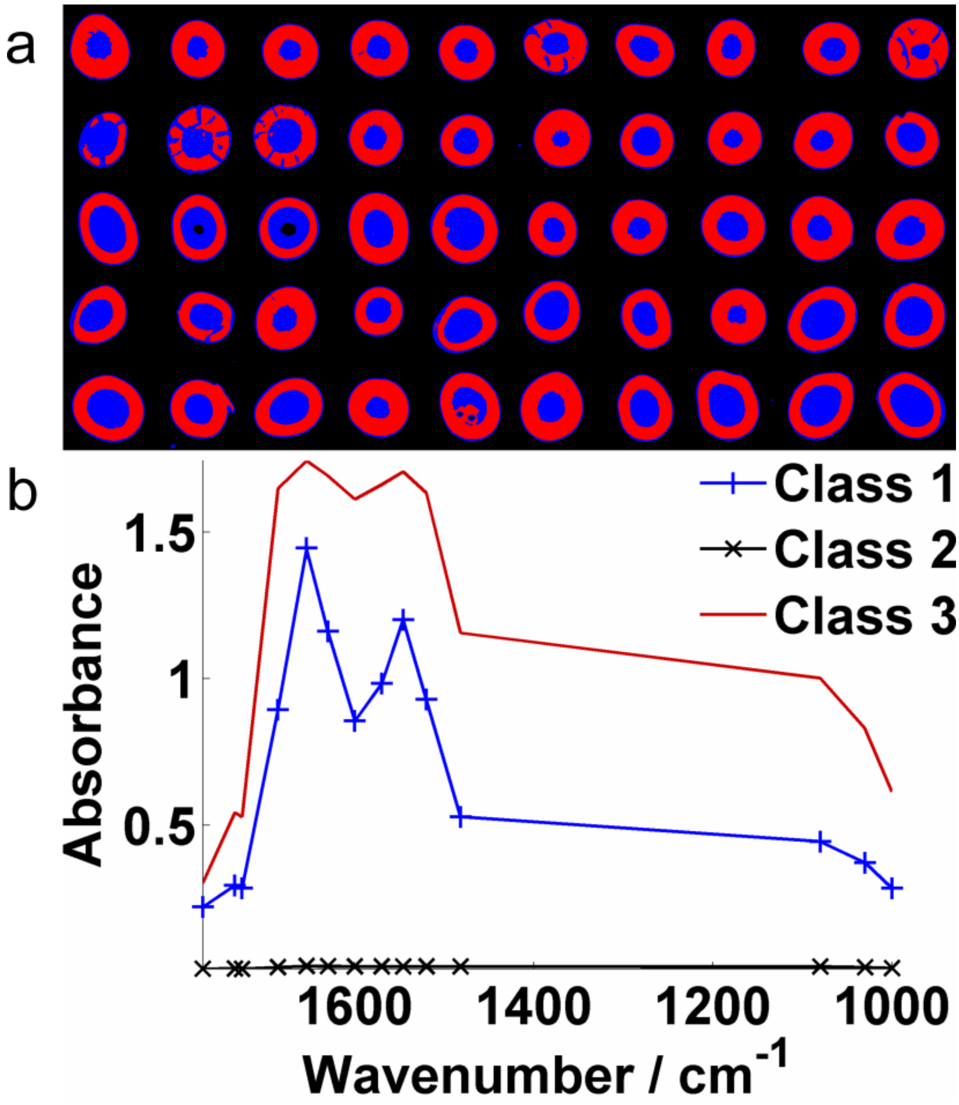

Supplement: Supplementary Information [file srep20173-s1.pdf]
